# Disaster Triage Skills Training: An Introductory Virtual Simulation for Medical Students

**DOI:** 10.7759/cureus.39417

**Published:** 2023-05-23

**Authors:** Kiran Pandit, Emma Healy, Raleigh Todman, Ashley Kingon, Melissa Wright, Marc Raymond, Jason Hill, John Jeffrey, Dimitrios Papanagnou, Christopher Tedeschi

**Affiliations:** 1 Emergency Medicine, Albert Einstein College of Medicine, New York, USA; 2 Emergency Medicine, Columbia University College of Physicians and Surgeons, New York, USA; 3 Center for Teaching and Learning, Columbia University, New York, USA; 4 Center for Engaged Pedagogy, Barnard College, New York, USA; 5 Emergency Medicine, Thomas Jefferson University, Philadelphia, USA

**Keywords:** emergency medicine, virtual, online, triage, medical education, disaster medicine, simulation

## Abstract

Background

Disaster triage training equips learners with the critical skills to rapidly evaluate patients, yet few medical schools include formal triage training in their curriculum. Simulation exercises can successfully teach triage skills, but few studies have specifically evaluated online simulation to teach these skills to medical students.

Aims

We sought to develop and evaluate a largely asynchronous activity for senior medical students to practice their triage skills in an online format.

Methods

We developed an online, interactive triage exercise for fourth-year medical students. For the exercise, the student participants acted as triage officers for an emergency department (ED) at a large tertiary care center during an outbreak of a severe respiratory illness. Following the exercise, a faculty member led a debriefing session using a structured debriefing guide. Pre- and post-test educational assessments used a five-point Likert scale to capture the helpfulness of the exercise and their self-reported pre- and post-competency in triage. Change in self-reported competency was analyzed for statistical significance and effect size.

Results

Since May 2021, 33 senior medical students have completed this simulation and pre- and post-test educational assessments. Most students found the exercise “very” or “extremely” helpful for learning, with a mean of 4.61 (SD: ±0.67). Most students rated their pre-exercise competency as “beginner” or “developing” and their post-exercise competency as “developing” or “proficient” on a four-point rubric. The average increase in self-reported competency was 1.17 points (SD: ±0.62), yielding a statistically significant difference (p < 0.001) and large effect size (Hedges’ g: 1.94).

Conclusions

We conclude that a virtual simulation can increase students’ sense of competence in triage skills, using fewer resources than in-person simulation of disaster triage. As a next step, the simulation and the source code are publicly available for anyone to engage with the simulation or adapt it for their respective learners.

## Introduction

The ability to recognize patients who require urgent or emergent care and initiate evaluation and management is a requirement for all graduating medical students [1]. Despite being a core competency for training, few medical schools have curricula that immerse trainees in learning experiences to practice the skill set of triage, defined as being able to make informed, time-sensitive clinical decisions with limited available resources. Mass casualty triage is an even more specific skill, one that students seldom experience as disasters are, by definition, rare and occur unpredictably. Simulation is one way for learners to gain exposure to this skill. While in situ disaster simulations are resource-intensive and often provide only one or two learners the opportunity to experience the role of a triage officer at a time, a virtual simulation may be a more sustainable educational option. We describe the development and evaluation of an online module that affords senior medical students the opportunity to practice and develop the skill of triaging patients during a mass casualty event, a critical skill as communities around the globe continue to face disasters causing significant morbidity and mortality. This project was initially presented as a poster at the meeting of the Council of Residency Directors in Emergency Medicine, San Diego, California, on March 29, 2022.

## Materials and methods

Development

This online triage exercise (Appendix A) was developed by a medical school faculty affiliated with a large, urban medical center in New York City, New York. Two emergency department (ED) physicians with experience in austere medicine and medical education developed the medical content. A grant received from the Office of the Provost provided educational design guidance and technical support from the institution’s center for teaching and learning to create the simulation. The simulation was written using an open-source code (Appendix B), as the objective was not only to provide the simulation experience to our students but also to allow learners all over the world to experience the simulation. We chose to use an open-source code to give instructors at our and other institutions the opportunity to adapt the simulation to fit their local context, to modify the simulated patient cases to reflect realistic scenarios specific to their locale, and to increase or decrease the complexity of the cases and decision-making to suit more advanced or more junior learners.

The learning objectives of the simulation were to 1) describe the role and responsibilities of the hospital triage officer during a disaster, 2) make rapid decisions for the clinical management of patients during a disaster (including emergency severity index {ESI} levels, ED location assignments, airway interventions, and the need for emergent consults or other interventions), 3) argue the clinical rationale for decisions made about emergent interventions (including resource constraints such as bed availability), 4) practice a balance of efficiency and effectiveness while rapidly triaging multiple patients during a disaster, and 5) recognize the challenge of triaging multiple patients in rapid succession in the context of incomplete information (such as the uncertainty of the potential needs of future patients).

As per the institutional guidelines, this work did not require submission to the institutional review board (IRB), as it is an evaluation of curricular elements of an educational program.

Participants

The inclusion criteria were medical students who have completed their preclinical years of training and major clinical rotations and have knowledge of the ESI levels and basic respiratory interventions. We implemented this online triage simulation with three separate groups of fourth-year medical students enrolled in an austere medicine elective (15 students in May 2021 and nine students in May 2022) or virtual emergency medicine elective (nine students in October 2021). The austere medicine elective is a one-month course that focuses on content related to wilderness medicine, environmental illness, and disaster preparedness and response while introducing students to overarching skills, such as improvisation, teamwork, and resource allocation. The virtual emergency medicine elective is a two-week course for emergency medicine-bound visiting medical students who have completed a one-month in-person emergency medicine rotation. This elective focuses on exposing students to the educational culture of the emergency medicine residency program through a combination of virtual patient care (urgent care telemedicine shifts), synchronous didactics (e.g., simulations, electrocardiogram {EKG} sessions, and residency conferences), and asynchronous content such as subspecialty discussion boards and independent learning with peer-to-peer feedback.

Setting and equipment

Students can conduct this simulation exercise entirely online and asynchronously in any web-based browser. Each participant requires a computer with internet access. The simulation is not designed to be used on mobile devices (tablets and smartphones) because it does not adjust to different screen sizes and viewports. No specific software platforms are necessary to run the simulation. The synchronous debrief session can occur either in person or over a virtual meeting platform and requires a facilitator skilled in disaster triage and debriefing.

Scenario template

We presented the simulation to students as an opportunity to practice their triage skills in a time-sensitive environment. The participants were sent instructions for how to complete the assignment (Appendix C) and were provided with a five-minute demonstration video (Appendix D) to become acquainted with the platform’s interface. They were instructed to complete the simulation only once to encourage genuine reactions to the stresses and challenges of making triage decisions about unknown patients under time pressure.

The triage simulation consists of four sequential steps: introduction, engagement, reflection, and summary. The introduction page establishes the scenario for the simulation, where each participant acts as a triage officer for an ED at a large urban, academic tertiary care center with all available subspecialties and intensive care unit (ICU) capacity during an outbreak of a severe respiratory illness. This page also provides instructions for the simulation.

**Figure 1:**
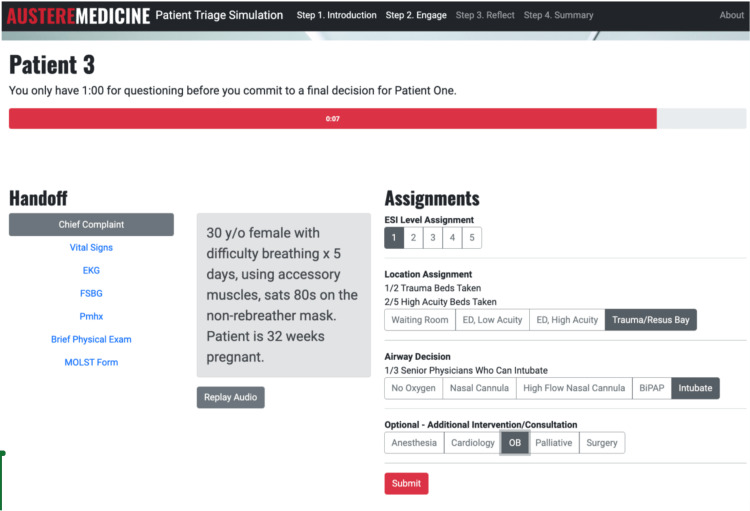
Example of a patient presentation during online triage simulation Accessed from https://austere-medicine.ctl.columbia.edu/triage EKG, electrocardiogram; FSBG, finger-stick blood glucose; Pmhx, past medical history; MOLST, medical orders for life-sustaining treatment; y/o, year old; sats, oxygen saturation; ESI, emergency severity index; ED, emergency department; resus, resuscitation; BiPAP, bilevel positive airway pressure; OB, obstetrics

In the engagement section, the simulation presents participants with 10 patients in rapid succession. Each patient case begins with an audio recording of an emergency medical technician (EMT) handoff that automatically plays (Figure 1).

The participants can request follow-up information selectively, including vital signs, EKG findings, finger-stick blood glucose levels, past medical history, brief physical examination findings, and medical orders for life-sustaining treatment (MOLST) form status. Students have limited time (ranging from 55 to 90 seconds) to collect data and make decisions. They must decide the patient’s ESI level (from 1 to 5), what respiratory intervention is required (ranging from no oxygen to intubation), if they should consult any subspecialty services (such as anesthesia, cardiology, obstetrics, palliative care, or surgery), and where to send the patient (waiting room, low-acuity ED, high-acuity ED, and trauma bay). In the simulation, the number of beds and senior physicians available to help with procedures is both limited.

**Figure 2:**
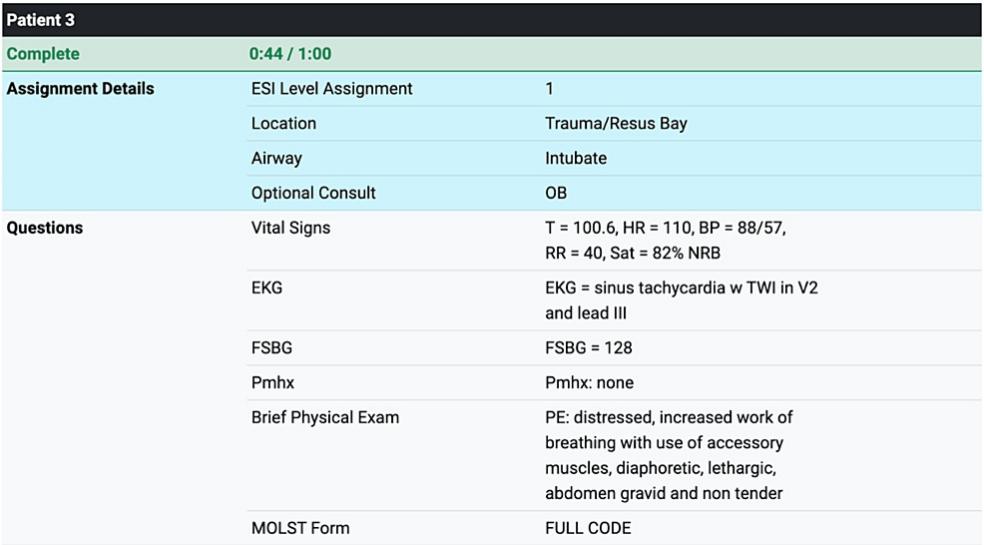
Example of patient triage simulation summary Accessed from https://austere-medicine.ctl.columbia.edu/triage ESI, emergency severity index; EKG, electrocardiogram; FSBG, finger-stick blood glucose; Pmhx, past medical history; MOLST, medical orders for life-sustaining treatment; resus, resuscitation; OB, obstetrics; T, temperature; HR, heart rate; BP, blood pressure; RR, respiratory rate; sat, oxygen saturation; NRB, non-rebreather face mask; TWI, T-wave inversion; PE, physical examination

In the reflection section, which immediately follows the 10 patient encounters, the participants are instructed to record brief reflections for each scenario. Afterward, in the summary section, a final page displays each patient’s full history, the participant’s selections from the simulations, and the reflections that were recorded after the simulation was completed (Figure 2). Students can download these summaries to be reviewed in the debrief session. 

Feedback and debriefing

For each group, one emergency medicine faculty member conducted a one-hour synchronous debrief via Zoom (Zoom Video Communications, Inc., San Jose, CA). The session was designed to examine how students felt while making rapid triage decisions, as well as to provide education on which triage decisions were most appropriate and why. A debriefing guide was created to provide faculty facilitators a framework for organizing the session (Appendix E). This guide encouraged facilitators to use the PEARLS framework, a four-phase process that encourages learner self-assessment, focuses discussion, and promotes the delivery of information as directive feedback [2]. In this framework, instructors were advised to begin the session with open-ended questions (e.g., “what are your initial reactions to the simulation?”) and to explore student reactions and emotions. Next, instructors encouraged learners to describe and analyze the key components from each case, exploring gaps in knowledge and opportunities for education. Finally, instructors concluded the session with a final summary to review take-home points. The debriefing document also provided an “answer key” that provides brief explanations of the learning points and most appropriate decisions for each case.

Implementation

This simulation exercise was assigned to medical students in the electives described above toward the middle of their elective rotation. For the austere medicine elective, the simulation was assigned during the week focused on hospital-based disaster response. Students were allotted several days to engage independently with the asynchronous portion, followed by the synchronous debrief.

As this was meant to be a formative learning experience for students, they were not summatively assessed for their performance during the simulation or debriefing process. All students completed a post-course educational assessment regarding the learning utility of the simulation and their self-reported confidence as a triage officer before and after the exercise (Appendix F). In this assessment, the self-reported helpfulness of the triage simulation for student learning was assessed using a five-point Likert scale from “not at all helpful” to “extremely helpful.” Self-reported competence as a triage officer was assessed using a four-point Likert scale that included beginner (“students do not have the ability to apply triage principles effectively in a mass casualty situation”), developing (“students are able to apply some triage principles to patients in a mass casualty situation but mistriage some patients”), proficient (“students are able to effectively apply triage principles to most patients in a mass casualty situation”), and advanced (“students are able to effectively apply triage principles to all patients in a mass casualty situation, under significant time pressure, and are able to explain rationales for their decisions”).

## Results

**Figure 3:**
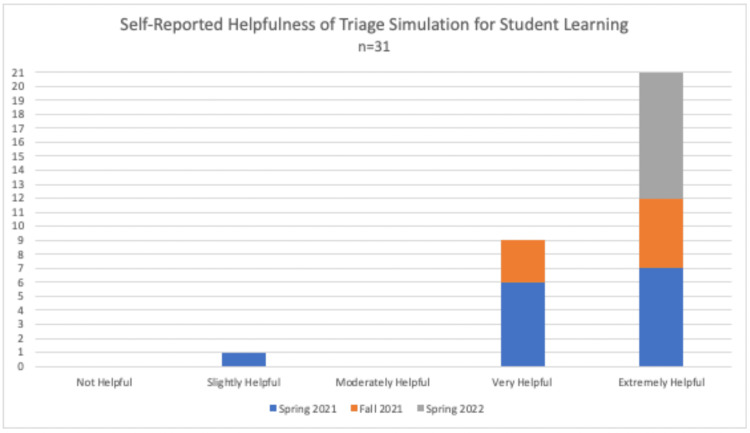
Self-reported helpfulness of triage simulation for student learning

A total of 31/33 students completed the simulation, debriefing session, and a post-simulation educational assessment (response rate: 94%) as a component of austere and emergency medicine electives. On a five-point Likert scale, most students (96.8%) found the exercise “very” or “extremely” helpful for learning, with a mean of 4.68 (SD: ±0.48) (Figure 3). Twenty-one students (67.7%) gave the highest score of 5 (“extremely helpful”).

**Figure 4:**
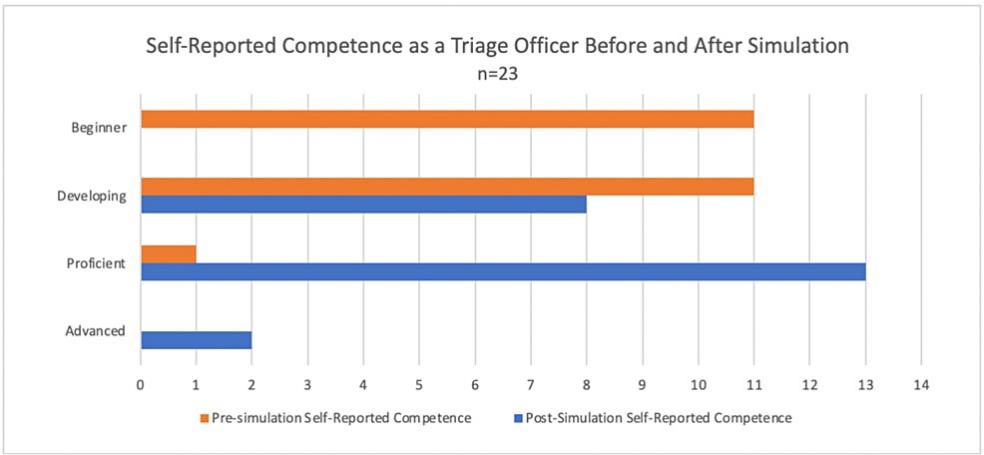
Self-reported competence as a triage officer before and after simulation

Twenty-three students who completed the simulation as part of an austere medicine elective in 2021 or 2022 also responded with their self-reported pre- and post-competency as a triage officer. Most students (95.7%) rated their pre-exercise competency as “beginner” or “developing” on a four-point rubric (Figure 4). Using the same rubric, most students (91.3%) rated their post-exercise competency as “developing” or “proficient.” The average increase in self-reported competency was 1.17 points (SD: ±0.62). Using a paired t-test, there was a statistically significant difference between the students’ self-reported competency scores pre- and post-simulation (p < 0.001) [3]. The effect size was large, with a Hedges’ g score of 1.94.

In their end-of-course evaluations, students were able to provide free-text comments about the simulation. Overall, they appreciated the opportunity to consider resource allocation, make time-sensitive decisions, and discuss the patient presentations after the session. Sample responses included the following: “I learned a lot about triage and managing high volumes of undifferentiated patients coming into the hospital during an MCI/pandemic…; it was extremely formative for understanding wilderness/environmental medicine, as well as emergency/hospital-based medicine,” “it was helpful to discuss the conditions and treatments needed for various patients and considerations for how to prioritize in limited-resource settings,” “these simulations prepared me for managing patients in resource-limited environments that include the hospitals/emergency rooms we work at,” and the simulations “[m]ade you really feel like you’re in the moment and have to make decisions on the fly.”

## Discussion

Being able to triage patients effectively during surge conditions is a critical skill for physicians and a foundational component of any disaster response effort. The United Nations (UN) 2015 Sendai Framework for Disaster Risk Reduction declared that developing the capacity of healthcare workers to understand disaster risk and implement risk reduction strategies is a crucial priority [4]. The ability to triage patients effectively is a core skill for physicians involved in medical emergencies, mass casualty incidents, and disasters and is fundamental to emergency medicine training programs [5]. Triage skills, however, are relevant to all medical specialties that may be called upon to assist with pandemics or other sources of patient surges. Surprisingly, few medical schools include formal triage or disaster preparedness training in their curriculum. A web-based survey disseminated among medical students in the United States found that a majority of respondents believed that they received inadequate disaster preparedness education [6]. Another survey completed by incoming interns at an academic teaching hospital discovered that only 47% of interns had received formal training in disaster preparedness during medical school [7]. A survey sent to administrators at each medical school in the United States accredited by the Association of American Medical Colleges (AAMC) showed an even lower percentage: only 31% of medical schools reported that they had incorporated disaster medicine into their curriculum [8].

Triage simulation exercises can effectively teach skills of rapid patient evaluation and decision-making [9-13]. Triage simulation, together with a structured debriefing, can result in improved triage accuracy in pediatric residents [9] and improved confidence in medical students [10]. As in situ simulation can be resource-intensive, a computerized simulation may be a more feasible alternative, and serious games are increasingly being used to augment simulation in medical education [14]. Virtual reality (VR) simulation has been shown to be equivalent to live simulation to test and improve medical student triage skills [11], and paramedic students using virtual reality were able to quickly and accurately triage simulated highway bus crash patients [12]. Virtual reality equipment may be cost-prohibitive, but online simulation overcomes resource barriers, is compatible with remote learning, and can be easily accessible and scalable. The computerized simulation of the triage of emergency department patients has been used to evaluate the triage performance of emergency department nurses and physicians [15,16]. Screen-based simulation of disaster triage has been previously demonstrated to improve field triage accuracy in prehospital providers [13,17]. Nursing students enjoyed learning triage skills via a web-based simulation of an earthquake and appreciated the immediate feedback, compared to learning by reading [18].

There remains a dearth of literature on the role of online simulation in teaching hospital-based disaster triage skills to medical students. As we describe in this report, we developed a largely asynchronous activity for senior medical students to practice triage skills during a disaster. Our simulation aims to develop the students’ competency to recognize patients who require urgent or emergent care and initiate management. We constructed the platform in such a way as to encourage participants to experience time pressure and practice assigning ESI level, location, consult services, and respiratory intervention. The simulation addresses an existing gap in many medical schools’ curricula, where students receive inadequate formal triage skills and disaster preparedness training [6-8]. Initial participants found that the resource was helpful for their learning, and there was a statistically significant increase in their self-reported competence as a triage officer after completing the exercise.

There are limitations in our assessment of the resource. The participants were not formally assessed for triage speed or accuracy before, during, or after the simulation; therefore, our comparisons of pre- and post-competency relied on self-reported measures. Self-reported competency was assessed shortly after participation; long-term impact was not assessed. The participants’ experiences with the virtual simulation were not correlated with their skills or comfort during in-person triage scenarios. There was no control group for comparison and no assessment of baseline triage performance for individual comparisons. Students may have bias based on the subject matter or their previous experience. Additionally, certain critical aspects of a real triage situation, such as teamwork, are difficult to integrate into a virtual simulation. Resource barriers were not evaluated. In terms of uptake outside our project, the exact numbers of hits and engagement with the simulation have not yet been tracked.

The tool expands the current field of simulation exercises by offering a free, online, asynchronous activity accessible by any institution or learner. The benefit of this virtual simulation is that participants can complete it from any location, without additional expense, addressing some of the resource limitations of live and VR simulations. This resource can feasibly be implemented at other institutions. The only requirements for implementation are that participants have a computer and internet access and that a facilitator trained in triage and debriefing conducts the post-simulation debrief session.

In addition to the simulation being freely accessible online, the source code is open, enabling anyone to develop their own adaptations to suit their local context. We plan for future development that will allow learners to select their experience level, offering different versions of the simulation with either simplified or complex cases, and we hope to track global engagement with our simulation.

## Conclusions

Online triage simulation is helpful for medical student learning and is associated with a statistically significant increase in the students’ self-reported competence in triage while addressing some of the resource barriers of in situ mass casualty simulation. The simulation is publicly available for any institution and any learner to use, and the source code for this simulation is also publicly available for anyone to adapt for their own learners.

## Appendices

Appendix A: Online triage simulation link

https://austere-medicine.ctl.columbia.edu/triage

Appendix B: Online triage simulation source code

https://github.com/ccnmtl/austere-med-interactives

Appendix C: Directions for participation

https://docs.google.com/document/d/19e6WTDcsiW8SDU0Li6bKrQTL-KB4q9Yk84xMZtKe2tc/edit#heading=h.g2ss5khcxnw6

Appendix D: Online triage simulation demonstration video

https://www.youtube.com/watch?v=PC_0zuCAqiY&feature=youtu.be&ab_channel=ColumbiaLearn

Appendix E: Debriefing materials

https://docs.google.com/document/d/1JaBfOCoQGwvHsQu4Uy9EXjYV7CsIvS9bW79eK-sHtwI/edit

Appendix F: Post-session educational assessment

https://docs.google.com/document/d/1K7zo2e_dcmsV4Gbw-0DuTjym0jV5ch4idB5T2RTStUY/edit
